# 3-Dimensional facial expression recognition in human using multi-points warping

**DOI:** 10.1186/s12859-019-3153-2

**Published:** 2019-12-02

**Authors:** Olalekan Agbolade, Azree Nazri, Razali Yaakob, Abdul Azim Ghani, Yoke Kqueen Cheah

**Affiliations:** 1 Department of Computer Science, Faculty of Computer Science & IT, Universiti Putra Malaysia, Serdang, Selangor Malaysia; 2 Department of Software Engineering, Faculty of Computer Science & IT, Universiti Putra Malaysia, Serdang, Selangor Malaysia; 3 Department of Biomedical Science, Faculty of Medicine and Health Sciences, Universiti Putra Malaysia, Serdang, Selangor Malaysia

**Keywords:** Facial expression recognition, 3D faces, Multi-point warping, Automatic facial landmark, PCA, LDA

## Abstract

**Background:**

Expression in *H-sapiens* plays a remarkable role when it comes to social communication. The identification of this expression by human beings is relatively easy and accurate. However, achieving the same result in 3D by machine remains a challenge in computer vision. This is due to the current challenges facing facial data acquisition in 3D; such as lack of homology and complex mathematical analysis for facial point digitization. This study proposes facial expression recognition in human with the application of Multi-points Warping for 3D facial landmark by building a template mesh as a reference object. This template mesh is thereby applied to each of the target mesh on Stirling/ESRC and Bosphorus datasets. The semi-landmarks are allowed to slide along tangents to the curves and surfaces until the bending energy between a template and a target form is minimal and localization error is assessed using Procrustes ANOVA. By using Principal Component Analysis (PCA) for feature selection, classification is done using Linear Discriminant Analysis (LDA).

**Result:**

The localization error is validated on the two datasets with superior performance over the state-of-the-art methods and variation in the expression is visualized using Principal Components (PCs). The deformations show various expression regions in the faces. The results indicate that Sad expression has the lowest recognition accuracy on both datasets. The classifier achieved a recognition accuracy of 99.58 and 99.32% on Stirling/ESRC and Bosphorus, respectively.

**Conclusion:**

The results demonstrate that the method is robust and in agreement with the state-of-the-art results.

## Background

Emotions in human face play a remarkable role when it comes to social communication. The identification of expressions by human beings is relatively easy and accurate. However, achieving the same result by machine remains a challenge in computer vision. Human face is the part that hosts the most crucial sensory organs. It also acts as the central interface for appearance, communication, expression and identification [1]. Therefore, acquiring its information digitally is important to researchers. This makes landmark-based geometric morphometrics methods for facial expression a new insight into patterns of biological emotion variations [2]. Many advances have been proposed in the area of acquisition of facial landmark but with several challenges especially in three-dimensional model. One of the challenges is the insufficient acquisition of 3D facial landmarks. Another challenge is the lack of homology due to manual annotation. Whereas complex mathematical analysis has made many works un-reproducible in 3D facial landmark acquisition.

The use of three-dimensional face images in morphometrics does not only give room to cover a wider area of human facial region but also retains all the geometric information of the object descriptors [3, 4]. In modality comparison, 3D face has higher detection rate than that of 2D due to its higher intensity modality [5]. Furthermore, during subjection to systematically increasing pitch and yaw rotation experiment performed in [6], there was a dropped in expression recognition performance in 2D while that of 3D remained constant. This is as a result of occlusion effects substantial distortion in out-of-plane rotations. More so, in the area of feature transformation and classification, 3D modality shows a little improvement with higher confidence over 2D. But in terms of depth features, both show the same performance; and the cost of 3D model in terms of processing is higher than that of 2D [5].

Below is the summary of the main contribution of this work:

1. We developed an approach for 3D facial landmark using multi-points warping. This approach has extended the computational deformation processing in [7] to improve the annotation performance using a less complex pipeline. We used six iterations and hundred to 5 % exponential decay sliding step in our method to ensure convergence and optimum smoothness.
2. Due to the easy detection, pose correction [8] and invariant to facial expression of nose tip [9], Pronasale was selected as the most robust and prominent landmark point. Since the nose tip area can be approximated as a semi-sphere of the human face. This determines the location where the sliding points begin to spread across the facial surface.
3. We have tested the method on two public 3D face databases (Stirling/ESRC and Bosphorus) to validate the precision of the annotation of the landmarks with the state-of-the-art methods.
4. We have validated the usability of our approach through its application to soft-tissue facial expression recognition in 3D. By using PCA for feature selection, we classify six expressions on both datasets. So far, to the best of our knowledge, sliding semi-landmark approach to facial landmarking has not been applied to solve problem relating to soft-tissue facial expression recognition in 3D.

**Fig. 1:**
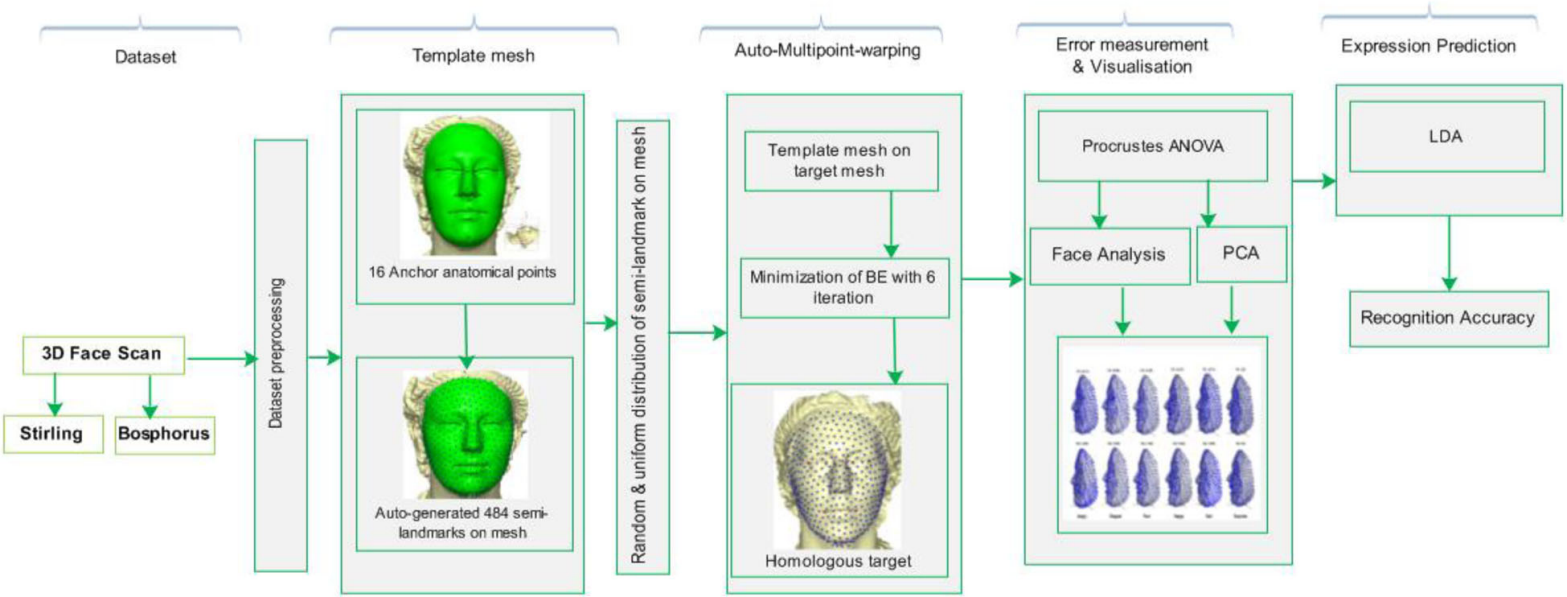
Architecture of the proposed method

Section one of this study focuses on the introduction, section two discusses the related studies. In section three, the implementation of the methodology is presented with supporting references where short explanation has been provided. Section four discusses the results of the implementations. In section five, a more detailed discussion is presented for the clarification of the result and comparison with state-of-the-art methods. The last section concludes the study and presents the limitations and future direction. Figure 1 shows the architectural diagram of the application of multi-points warping to the analysis of facial expression recognition in 3D.

## Literature review

The term “Morphometrics” was coined by Robert E. Blackith more than 50 years ago, who applied multivariate statistical methods to the basic carapace morphology of grasshoppers [10]. Morphometrics is the study of shape variation and its covariation with other variables [7, 11]. According to DC Adams, et al. [12], morphometrics was traditionally the application of multivariate statistical analyses to a sets of quantitative variables such as length, width, height and angle. But advances in morphometrics have shifted focus to the Cartesian coordinates of anatomical points that might be used to define more traditional measurements. Morphometrics examines shape variation, group differences in shape, the central tendency of shape, and associations of shape with extrinsic factors [13]. This is directly based on the digitized x,y, (z)-coordinate positions of landmarks, points representing the spatial positions of putatively homologous structures in two or three dimensions; whereas conventional morphometric studies utilize distances as variables [7, 11, 14]. The landmark was described in LF Marcus, et al. [15] as a point in a bi- or three-dimensional space that corresponds to the position of a particular trait in an object. This set of points, one on each form, are operationally defined on an individual by local anatomical features and must be consistent with some hypothesis of biological homology. But the formal landmark definitions were provided by anthropometric studies in [16]. This work by LG Farkas [16] has been provided as the standard for head and face landmark definitions through the study of thousands of subjects from different races. These have produced a large number of anthropometric studies in the head and face regions.

A flexible and mathematically rigorous interpolation technique of D’Arcy Thompson’s transformation grids [17], called Thin Plate-Spline (TPS), was brought into morphometrics. This ensures that the corresponding points of the starting and target form appear precisely in corresponding positions in relation to the transformed and untransformed grids [18]. With the application of Iterative Closest Point (ICP), landmark correspondence can iteratively be registered in the vicinity of a landmark with a re-weighted error function. Morphometrically, some studies have been proposed which computed localization errors of facial landmarks on Bosphorus dataset. A novel 3D constrained Local Models (CLM) approach facial landmark detection in 3D images is proposed in [19], which capitalizes on the Independent Component Analysis (ICA) properties in order to define appropriate face Point Distribution Model (PDM) tailored to the mesh manifold modality. Each sample contains 24 manually annotated facial landmarks. While the PDM includes 33 landmarks and 14 of them are part of the ground truth set tested on Bosphorus database. An automatic method for facial landmark localization relying on geometrical properties of 3D facial surface was proposed in [20], working on complete faces displaying different emotions and in presence of occlusions. The method extracts the landmark one-by-one. While the geometrical condition remains unchanged, the method double-checks to ascertain whether pronasale, nasion and alare are correctly localized, otherwise the process starts afresh. The method is deterministic and is backboned by a thresholding technique designed by studying the behavior of each geometrical descriptor in correspondence to the locus of each landmark, experimented on Bosphorus database.

Though facial landmarks are known to be specific points with an anatomical meaning which has been described in Table 1; since a considerable amount of biological variability cannot be assessed using only anatomical landmarks [21], in order to quantify complex shapes, sliding semi-landmarks have been developed which can be placed on surfaces [22] or curves [7, 22]. This approach generates landmarks that are spatially homologous after sliding [23] which may be optimized by minimizing bending energy [24, 25] or Procrustes distance [26, 27]. Since sliding semi-landmarks have not been implemented in analysing facial expression for soft-tissue in 3D, we have decided to investigate the expression recognition using the application of multi-points warping approach.

**Table 1:** Procrustes ANOVAs for facial shape on Stirling and Bosphorus datasets

Effect	SS	MS	DF	F	P
Stirling					
Expression	0.178563	2.39E-05	7465	8.36	<.0001
Individual	1.02071	2.86E-06	356,827	1.86	<.0001
Error	0.041283	1.54E-06	26,874		
Bosphorus					
Expression	0.308295	4.13E-05	7465	16.4	<.0001
Individual	0.65404	2.52E-06	259,782	0.72	1
Error	0.09455	3.52E-06	26,874		

Emotion or expression recognition using facial analysis has been the current trend in computer vision but the diversity of human facial expression has made the emotion recognition somehow difficult [28]. Moreover, asides unidentifiable lighting challenges, the fairly significant differences in age, skin colour and appearance of individual placed additional burden on machine learning. When face subjects are transformed into feature vectors, any classifier can be used for expression recognition such as neural network, support vector machines, random forest, linear discriminant analysis, etc. But the uniqueness is the application of facial image information [29]. Due to the sensitivity of the change in head posture and illumination, the use of static 2D image is unstable for expression recognition. The use of 3D does not only play safe in the area of illumination and pose change but also enables the use of more image information. This is because facial expressions are generated by facial muscle contractions. It results in temporary facial deformations in both texture and facial geometry which is detectable in 3D and 4D [30]. The same successes achieved in 3D face recognition could still be naturally adopted for expression recognition [31]. According to M Pantic and LJ Rothkrantz [32] on facial expression analyser, facial expression follows the general properties for solving computer vision problems: face detection, landmark localisation, recognition or classification. As 3D databases are becoming more and more available in the computer vision community, different methods are being proposed to tackle the challenges facing facial expression recognition. Most of these studies are based on six fundamental expression classes or less: anger, fear, disgust, sadness, happiness, and surprise [33]. Many also focus on the use of local features which retrieves the topological and geometrical properties of the face expression [29, 34].

Linear discriminant analysis and many other classifiers have been used for classification in many face expression recognitions. A learn sparse features from spatio-temporal local cuboids extracted from human face was proposed in [35]. This has application of conditional random field classifiers for training and testing the model. In H Tang and TS Huang [36], similar distance feature was explored using automatic feature selection technique. This was done by maximizing the average relative entropy of marginalized class-conditional feature distributions. Using 83 landmarks, less than 30 features were selected. The features distance are subtracted from the features of the expressive scan on the neutral scan which they classified by Naive Bayes, Neural network and Linear Discriminant Analysis on BU-3DFE dataset. To approximate the continuous surface at each vertex of an input mesh, YL Wang Jun, Wei Xiaozhou, Sun Yi [6] proposed a cubic-order polynomial functions. It estimated coefficient at a particular vertex, formed the weingarten matrix for the local surface path. The eigenvectors and eigenvalues of the matrix could be derived by normal direction along the gradient magnitude. The facial region was described using 64 landmarks to overcome the lack of correspondence between the meshes. Their best performance was obtained using LDA; no rigid transformation is required due to the geometrical invariance of curvature-based features. To deal with issue of deformation of facial geometry which results from expression changes, C Li and A Barreto [37] proposed a framework that is composed of three subsystems: expressional face recognition system, neutral face recognition system and expression recognition system. This was tested on 30 subjects and was classified using LDA, but used only two expression groups.

H Li, et al. [38] proposed a novel method using fine-grained matching of 3D key-point descriptors by extending the SIFT-like matching framework to mesh data. To account average for reconstruction error of probe face descriptors, multi-task sparse representation algorithm was used. The approach was evaluated on Bosphorus database for expression recognition, pose invariant and occlusion. A comprehensive comparative evaluation was performed on Gavab, UND/FRGC, and Bosphorus in [39] by using local shape descriptor. The method captured distinguishing traits on the face by extracting 3D key-points. Similarity expression on faces was evaluated by comparing local shape descriptors across inlier pairs of matching key-points between gallery scans and probe. Using a Key-point-based Multiple Triangle Statistics (KMTS) with a Two-Phase Weighted Collaborative Representation Classification (TPWCRC), a robust to partial data, large facial expression and pose variations was proposed in [40]. The method was experimented on six databases including Bosphorus which achieved a promising result on occlusions, pose variation and expressions. A 3D face augmentation technique was proposed in [41], which synthesizes a number of different facial expressions from a single 3D face scan. The method showed excellent performance on BU-3DFE, 3D-TEC, and Bosphorus datasets, without application of hand-crafted features. A novel geometric framework for analysing 3D faces was proposed in [42] with the goals of averaging face shapes and comparing matching. The method presented facial surfaces by radial curves emanating from the nose tips, which was experimented on FRGCv2, GavabDB, and Bosphorus.

Furthermore, in order to address the issue of 2D counterpart and the handling of large intra-class and inter-class variability for human facial expression, W Hariri, et al. [43] proposed the use of covariance matrices of descriptors rather than using the descriptors themselves. Their work focused on application of manifold-based classification which was tested on BU-3DFE and Bosphorus databases. While extended local binary patterns was proposed in [44] for facial expression recognition from 3D depth map images where the results on Bosphorus showed better performance by the combination of 3D and 3D curvature.

## Experiment results

After the step-by-step methods in facial surface deformation of semi-landmark, the error assessment, the analysis, visualisation and classification of the experiment were performed using MorphoJ 1.06d [45], PAST 3.0 [46] and R 5.1 [47].

### Landmarks significance

The use of landmarks evolves when locating biological or anatomical features on human faces. Its validity is drawn from the morphometric analysis which depends on the biological justification for designation of the landmarks as stated in [3]. But not all the facial anatomical landmarks always indicate a meaningful significant measure. On Stirling dataset, the overall landmarks are tested using one way ANOVA to see the significant of the variation on each expression group, each group having the same degree of freedom (df = 1499). Angry: *F* = 133.9, *p* < 0.00001; Disgust: *F* = 120.9, *p* < 0.00001; Fear: *F* = 132.9, *p* < 0.00001; Sad: *F* = 130.2, *p* < 0.00001; Happy: *F* = 184.3, *p* < 0.00001; and Surprise: *F* = 117, *p* < 0.00001. Subsequently, same test was computed for Bosphorus on each expression group, each group having the same degree of freedom (df = 1499). Angry: *F* = 2507, *p* < 0.00001; Disgust: *F* = 1552, *p* < 0.00001; Fear: *F* = 3899, *p* < 0.00001; Sad: *F* = 2543, *p* < 0.00001; Happy: *F* = 2435, *p* < 0.00001; and Surprise: *F* = 1582, *p* < 0.00001. Furthermore, we conducted PERMANOVA (Non-Parametric MANOVA) which is a non-parametric test of the significant difference between the expression groups, based on the distance measured [48] with *F* = 7.76 and *P* = 0.0001 for Stirling dataset and *F* = 115.5 and *P* = 0.0001 for Bosphorus dataset. The large positive of F value indicates that there is a significant difference between the expression groups.

### Procrustes ANOVA

For the assessment of localization errors of the landmarks; the deviations of each landmark is obtained by simply calculating the amount of displacement from the average position calculated from all digitization and the variation accounts for the smallest portion of the total variation using Procrustes ANOVA. The localization errors accounts for only 0.041 and 0.095 for Stirling and Bosphorus, respectively, from the total variation (Table 1).

### PCA results

**Fig. 2:**
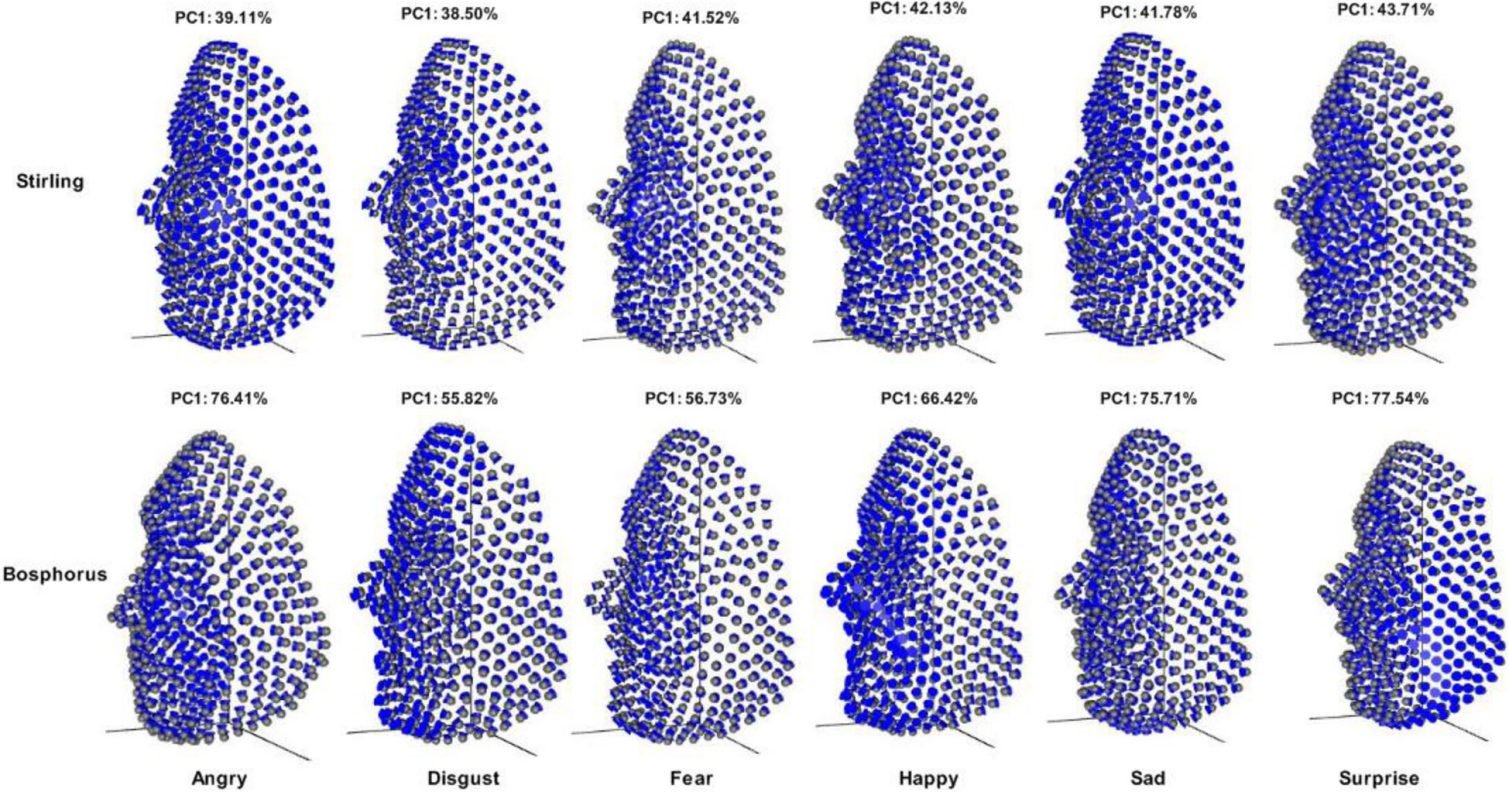
Visualisation of the expression group. PC deformation and the percentage variance of selected principal components, showing only the first principal component which accounted for the largest variation on both datasets

The PCA of the total sample of Stirling yielded 239PCs while Bosphorus yielded 179PCs. When each expression group was separately computed, each yielded 39PCs and 29PCs for Stirling and Bosphorus, respectively, all with non-zero variability. Using a broken stick approach of PCA selection [17], only first 2PCs in each group accounted for more than 58% of the shape variation for Stirling while only the first 2PCs accounted for more than 70% for Bosphorus. For Stirling: Angry (PC1: 39.11%, PC2: 18.99%), Disgust (PC1: 38.50%, PC2: 15.65%), Fear (PC1:41.52%, PC2: 17.51%), Sad (PC1: 41.78%, PC2: 17.51%), Surprise (PC1: 43.71%, PC2: 15.69%), and Happy (PC1: 42.13%, PC2: 16.96%). For Bosphorus: Angry (PC1: 76.41%, PC2: 8.96%), Disgust (PC1: 55.82%, PC2: 19.88%), Fear (PC1:56.73%, PC2: 13.28%), Sad (PC1: 75.71%, PC2: 8.99%), Surprise (PC1: 77.54%, PC2: 7.95%), and Happy (PC1: 66.42%, PC2: 9.09%). For the sake of visualisation, we only presented the deformations of the first PC which accounted for the largest variation after Procrustes fit in each expression group (Fig. 2) as 3D vectors away from the mean configuration [11].

### Classification

**Fig. 3:**
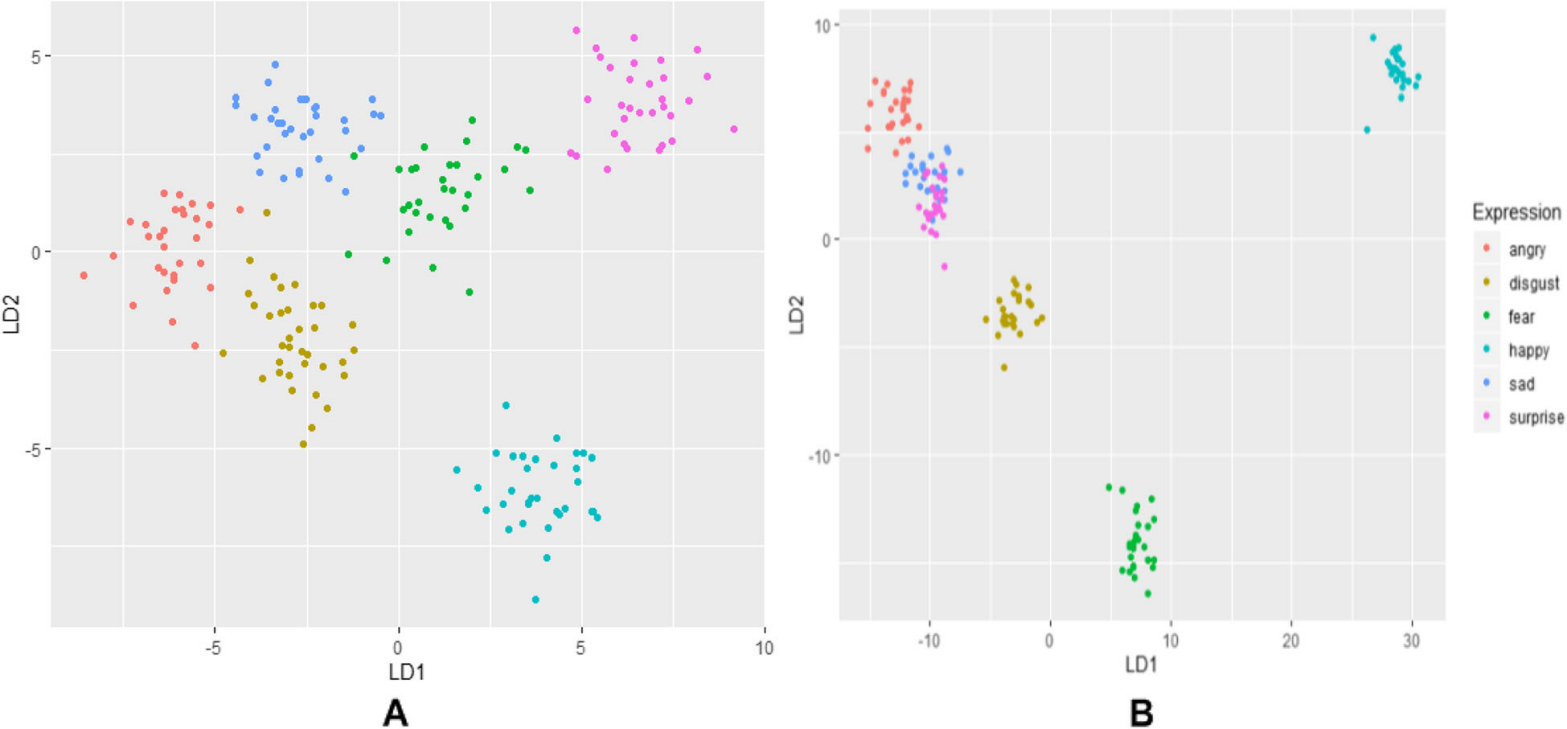
Scatterplot of Expression group. Separability and distribution of expression group using scatter plot. **a** Stirling dataset. **b** Bosphorus dataset

We used LDA to classify the expression variations of 240 sample faces of six different classes using 135 selected PCs in Stirling dataset and 180 sample faces of six different classes using 98 selected PCs in Bosphorus dataset. Since LDA is easy to implement and no tuning parameters or adjustment required which has successfully been applied to many previous studies [49, 50], etc. By using leave-one-out cross validation, the data was learned with 70% training and 30% testing. A call to LDA returned the prior probability of each expression class, the group means for each covariate, the coefficient for each linear discriminant (for the six classes, we have five linear discriminants) and the singular values that produced the ratio of the within-class and between-class standard deviation on the first two LDs variables returned the proportions of the variance by Stirling (LD1 = 36.23%, LD2 = 29.57%) and by Bosphorus (LD1 = 36.23%, LD2 = 29.57%) (Fig. 3). The confusion matrixes for both Stirling and Bosphorus are also produced in Table 2 and Table 3, respectively. These indicate that only Sad expression is slightly misclassified with 2.44% for Fear expression in Stirling dataset while only the same Sad expression is slightly misclassified with 4.55% for Surprise in Bosphorus dataset.

**Table 2:** Confusion matrix for six group facial expression recognition on Stirling dataset

%	Ang	Dis	Fea	Sad	Hap	Sur
Ang	100	0	0	0	0	0
Dis	0	100	0	0	0	0
Fea	0	0	100	0	0	0
Sad	0	0	2.44	97.56	0	0
Hap	0	0	0	0	100	0
Sur	0	0	0	0	0	100

**Table 3:** Confusion matrix for six group facial expression recognition in Bosphorus dataset

%	Ang	Dis	Fea	Hap	Sad	Sur
Ang	100	0	0	0	0	0
Dis	0	100	0	0	0	0
Fea	0	0	100	0	0	0
Hap	0	0	0	100	0	0
Sad	0	0	0	0	95.45	4.55
Sur	0	0	0	0	0	100

### LDA model performance

In this scheme, the dataset was divided into 70% training and 30% testing for both Stirling and Bosphorus. The scheme performance was measured using precision, recall and specificity.

1 $$ Sensitivity/ Recall= TP/\left( TP+ FN\right)\times 100 $$

2 $$ Specificity= TN/\left( FP+ TN\right)\times 100 $$

3 $$ Accuracy= TP+ TN/\left( TP+ FP+ TN+ FN\right)\times 100 $$

Where TP is the true positive, TN is true negative, FP is false positive, FN is false negative. The accuracy shows overall prediction performance; sensitivity is the capacity of features to accurately recognize an expression while specificity is the feature capacity to recognise a true expression. The classifier produced the percentage precision, sensitivity, specificity and accuracy of 99.70, 99.60, 99.90 and 99.58%, respectively for Stirling dataset and 99.20, 99.30, 99.90 and 99.32%, respectively for Bosphorus dataset. The performance metrics are displayed in Table 4, showing precision, recall and specificity.

**Table 4:** Performance metrics reports for facial expression on Stirling and Bosphorus dataset

	Stirling Dataset			Bosphorus Dataset		
Exp	Precision	Sensitivity	Specificity	Precision	Sensitivity	Specificity
Ang	1	1	1	1	1	1
Dis	1	1	1	1	1	1
Fea	1	0.975	1	1	1	1
Sad	0.976	1	0.995	0.954	1	0.992
Hap	1	1	1	1	1	1
Sur	1	1	1	1	0.96	1
Avg/Total	0.997	0.996	0.999	0.992	0.993	0.999

## Discussions

The Procrustes ANOVA suggests a modest but appreciable variation in facial shape. Shape differences are statistically significant even after averaging faces within expression. Small localization errors for both datasets show that the landmarks can be annotated with precision using the proposed method. Table 5 demonstrated superiority of our method on localization error when compared with state-of-the-art methods. Though, many approaches are available in addressing measurement error. Discussing such at length is beyond the scope of this study, more and extended details can be found in [51]. The expression recognition accuracy demonstrated superiority when compared with state-of-the-art methods (Table 6 and Table 7).

**Table 5:** Comparison of mean localization error with state-of-the-art method on Bosphorus datasets

Author	Method	Landmark	Mean error (mm)
[19]	CLM-ICA-GGD	33	2.71
[20]	Geometric Descriptor	13	4.75
This work	Multi-points warping	500	0.094

**Table 6:** Comparison of classification rates with state-of-the-art method on Stirling and Bosphorus datasets

Author	Method	Dataset	Classifier	Accuracy (%)
[39]	Local shape descriptor	Bosphorus	RANSAC	93.40
[40]	KMTS	Bosphorus	TPWCRC	98.90
[41]	Face augmentation technique	Bosphorus	CNN	99.20
[42]	Geometric framework	Bosphorus	–	87.06
[38]	Extended SIFT-like matching	Bosphorus	–	98.80
[6]	3D-PSFD	–	LDA	83.60
[52]	Differential Evolution based optimization	Bosphorus	SVM	84.00
[44]	Extended LBP	Bosphorus	SVM	76.98
[43]	Covariance matrices of descriptors	Bosphorus	SVM	86.17
This work	Multi-points warping	Stirling/ESRC	LDA	99.58
		Bosphorus	LDA	99.32

**Table 7:** Comparison of classification rates by each expression with state-of-the-art method on Bosphorus datasets

Author	Hap (%)	Fea (%)	Dis (%)	Ang (%)	Sad (%)	Sur (%)	Overall (%)
[52]	97.50	86.25	90.00	82.50	67.50	83.75	84.10
[43]	93.00	81.00	85.25	86.25	79.75	90.50	86.17
This work	100	100	100	100	95.45	100	99.32

There is agreement and consistency in our work with most of the state-of-the-art studies, which carried out similar work on Bosphorus dataset using different methods. In [52], a differential evolution based optimization was presented by first transforming 3D faces in to 2D plane using conformal mapping and selecting optimal features using Speed Up Robust Features (SURF). The method was tested on Bosphorus dataset and classified by SVM containing six basic expressions. The results indicated that Sad expression has the lowest recognition accuracy of 67.50%. The use of covariance matrices of descriptors proposed in [43] tested on Bosphorus dataset indicted that Sad expression has the lowest recognition rate of 79.75%. Though both results are in agreement with our study, yet our method performed better in the Sad expression with recognition rate of 95.45% on Bosphorus dataset.

The scatter plot of the expressions along the first two linear discriminants produced maximal separation between all groups; these linear discriminants are linear combinations of the original variables as in principal component analysis, which indicates amount of variation explained by these linear discriminants. The classifier classified the expression groups with accuracy of 99.58 and 99.32% for both Stirling and Bosphorus, respectively. Though some Sad faces were misclassified as Fear faces in Stirling dataset. This indicates that it is possible to misrepresent a Sad expression with Fear expression. While Sad faces were misclassified as Surprise in Bosphorus dataset. This also indicates that it is possible to misrepresent a Sad expression with Surprise expression. In the visualization of the expression using PCs, the deformations show various expression regions in the faces. In Stirling dataset, Surprise shows more expression in mouth region, Happy shows more expression in the cheek region, Angry and Disgust show more expression both in mouth and eyes regions. Only Sad seems to be very close to the neutral expression but slightly show expression in the whole facial regions. Whereas in Bosphorus dataset, Surprise shows more expression in cheek region, Sad and Fear show more expression both in mouth and eyes regions, Angry show more expression in the cheek region. While Happy and Disgust show more expression in the whole facial region.

To the best of our knowledge, there is currently no facial landmark annotation analysis and expression recognition performed using Stirling/ESRC dataset. Therefore, this is the first facial expression study using Stirling/ESRC dataset. According to T Fang, et al. [29, 53] who reported that additional 3D datasets in expression recognition with different modalities, plus some examples of spontaneous and natural behaviour captured in 3D are needed for researchers to evaluate their methods. We believe that, in the future this dataset will be used for many research benchmarks especially in the field of facial expression in 3D.

We strongly advise not to rely on broken stick of scree plot decision on PCA when it comes to classification or machine learning, further data wrangling must be performed. Note also that the features were never standardised during learning as the data has already been Procrustes-fitted in PAST software, as covariance matrix is always affected when such happens. Whereas there is no effect on covariance matrix for mean centering and variables scaling.

## Conclusions

This method combines pragmatic solutions to configure an optimized pipeline for high-throughput multi-points facial signature in three-dimensional. Only the reference surfaces and curves were warped to each sample faces using automatic warping approach and the errors were assessed using Procrustes ANOVA. The result acquired was further used in the selection of features for classification using PCA; and LDA was used to classified expressions. Such a high-throughput and accurate phenotypic facial data like this is not only valuable for facial expression recognition but also in forensic studies of human facial morphology, sexual dimorphism, anthropology, disease diagnosis and prediction, statistical shape or image analysis, face recognition and age estimation. In the feature, the method can be further improved by automatically applying the reference model to all the targets at once without applying to each target one after the other. Furthermore, ViewBox 4.0 does not work well in the annotation of eyeball when the eyes are opened. Though it does not affect the annotation and measurement of endocanthion and exocanthion as they lie at the tissue edges of the eyeballs; this will be addressed in the future studies.

## Methods

### Dataset & Description

The first dataset is acquired from Stirling/ESRC 3D face database captured by a Di3D camera system [54]. The image format used for this study is in wavefront obj file containing 240 faces which were randomly selected from different expression positions: Angry (40), Disgust (40), Fear (40), Happy (40), Sad (40), and Surprise (40). This is intended to facilitate research in sexual dimorphism, face recognition, facial expression recognition and perception. The dataset is being used as a test set for a competition on 3D face reconstruction from 2D images, with the 3D scans acting as ‘ground truth’ in IEEE conference. The second dataset is the Bosphorus database, which was intended for research on 3D and 2D human face processing tasks. A total of 180 subjects are rondomly selected for this study: Angry (30), Disgust (30), Fear (30), Happy (30), Sad (30), and Surprise (30). The dataset was acquired using structured-light based 3D system. The subjects were instructed to sit at a 1.5 m distance with sensor resolution in x, y and z depth of 0.3 mm, 0.3 mm, and 0.4 mm, respectively, with a high-resolution color texture [5, 55].

### Creating template mesh

**Fig. 4:**
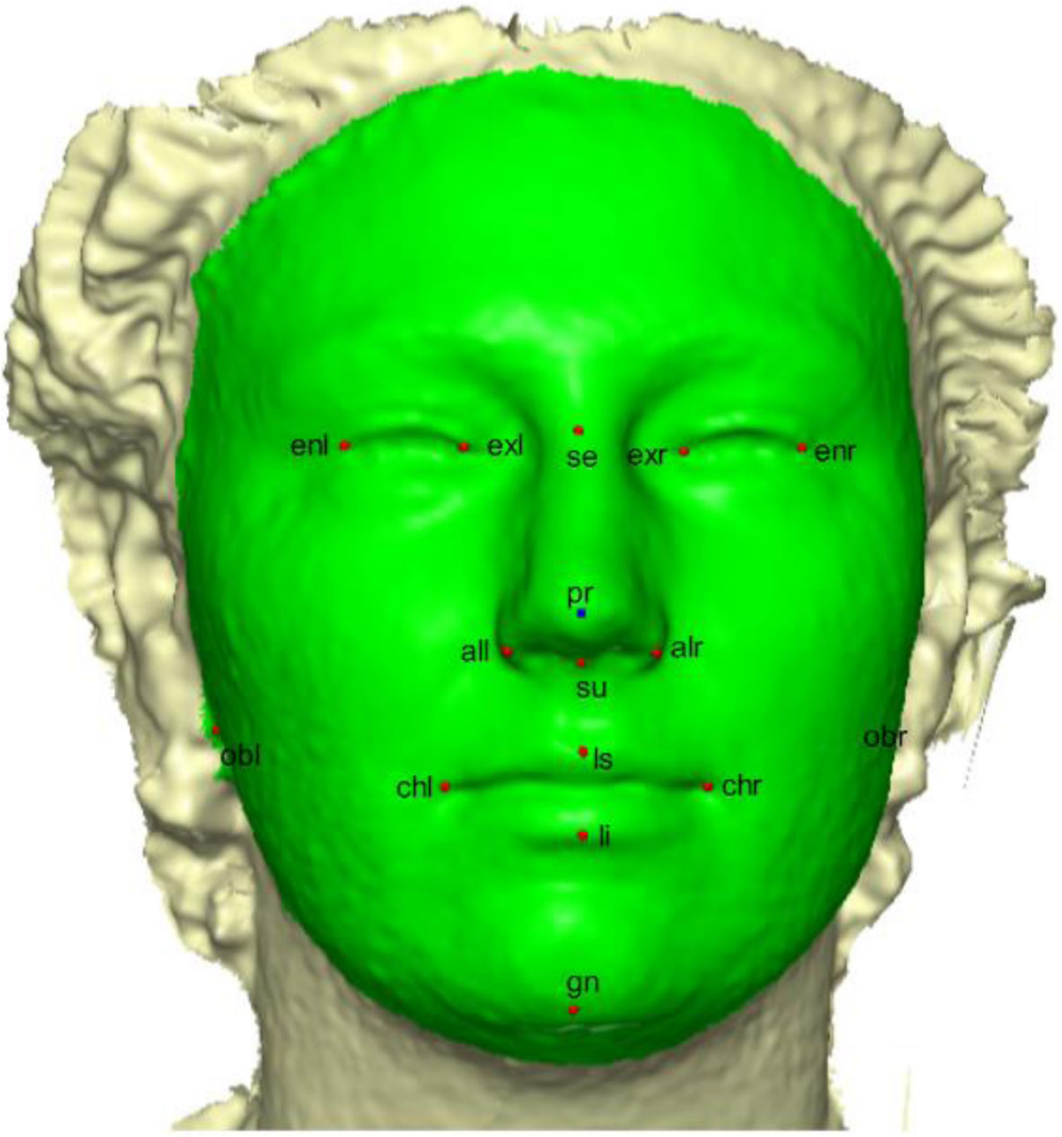
A three-dimensional mesh template with the location of the prominent point at the center of the face for pose-invariant correction. The 16 fixed anatomical landmarks are shown in red color. The blue color on the Pronasale indicates the point where the semi-landmarks begin the sliding process

**Fig. 5:**
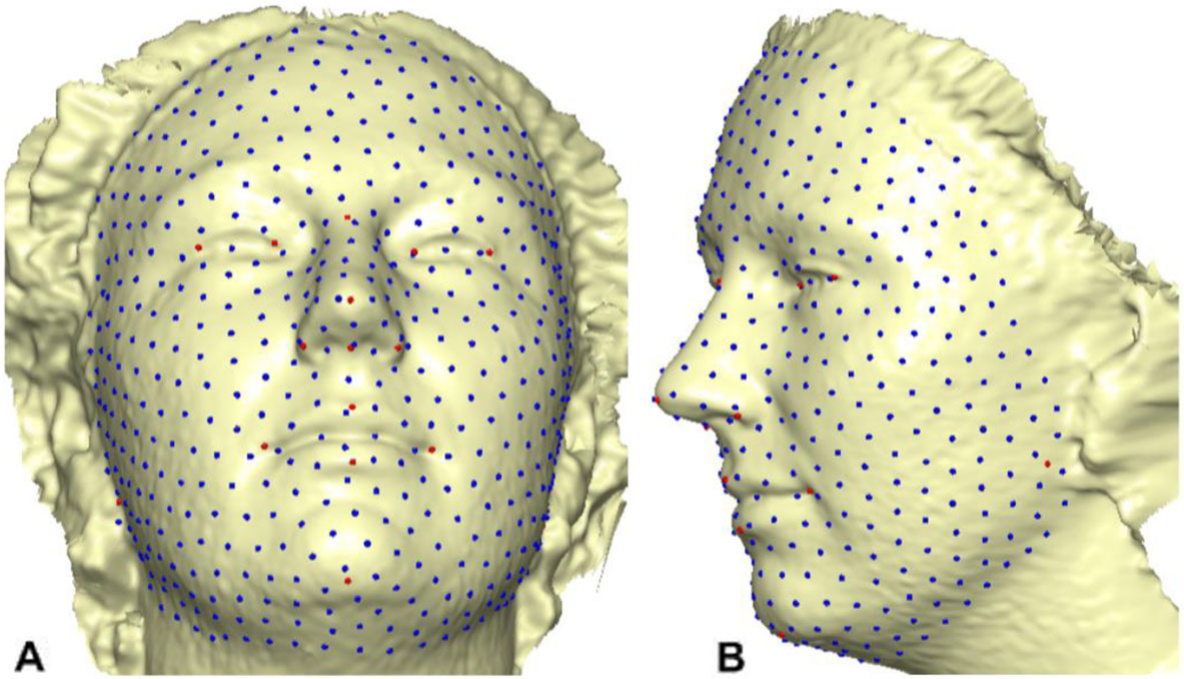
A three-dimensional mesh template with 500 landmarks for reference model. Showing 16 fixed anatomical points and 484 semi-landmarks with 1.5 mm radius. **a** Frontal skewed view. **b** Profile view

The template mesh was created by manually locating sixteen anatomical points on a 3D face (Fig. 4) with neutral expression called fixed points according to facial landmark standard [56] (details in Table 8). The anchor landmarks were not subjected to sliding but were used for establishing the warping fields that would be used for minimizing the bending energy. Due to the easy detection, pose correction [8] and invariant to facial expression of nose tip [9], Pronasale has been selected as the most robust and prominent landmark point. Since the nose tip area can be approximated as a semi-sphere of the human face. This is where the sliding points begin to spread across the facial surface. Using this anchor point (Pronasale), 484 semi-landmarks were automatically generated overlapping on Pronasale showing in blue color. These were first randomly placed on the facial mesh before they were uniformly distributed on the selected facial surface using the locational position of the anchor anatomical points with 1.5 mm radius to accommodate all the 500 points (see Additional file 1: Table S1 and Additional file 2: Table S2) (Fig. 5). To quantify the morphological data of a complex, three-dimensional trait of both reference and target shapes, we have used geometric morphometric tools based on a landmark-based methodology in [57–61] and the landmark acquisition process was fully implemented in ViewBox 4.0 [61].

**Table 8:** Anchor anatomical points and descriptions

No	Anchor Landmarks	3D Notation	Description
1	Endocanthion left	enl	Left most medial point of the palpebral fissure, at the inner commissure of the eye
2	Exocanthion left	exl	Left most lateral point of the palpebral fissure, at the outer commissure of the eye
3	Exocanthion right	exr	Right most lateral point of the palpebral fissure, at the outer commissure of the eye
4	Endocanthion right	enr	Right most medial point of the palpebral fissure, at the inner commissure of the eye
5	Sellion	se	Deepest midline point of the nasofronal angle
6	Pronasale	pr	The most anteriorly protruded point of the apex nasi
7	subnasale	su	Median point at the junction between the lower border of the nasal septum and the philtrum area
8	Alare left	all	Left most lateral point on the nasal ala
9	Alare right	alr	Right most lateral point on the nasal ala
10	Cheilion left	chl	Left outer corners of the mouth where the outer edges of the upper and lower vermilions meet
11	Cheilion right	chr	Right outer corners of the mouth where the outer edges of the upper and lower vermilions meet
12	Labiale superius	ls	Midpoint of the vermilion border of the upper lip
13	Labiale inferius	li	Midpoint of the vermilion border of the lower lip
14	Gnathion	gn	Median point halfway between pogonion and menton
15	Obelion left	obl	Left median point where the sagittal suture intersects with a transverse line connecting parietal foramina
16	Obelion right	obr	Right median point where the sagittal suture intersects with a transverse line connecting parietal foramina

### Multi-points warping

The geometry of curves and surfaces is easier in 2D or 3D but it is not so easy to define semi-landmarks for non-planar surfaces in 3D [62]. This is because they are not guaranteed to be homologous after first placement. This could alternately be achieved by subjecting the semi-landmark to sliding in the direction that reduces shape variance. This closely positions the points on the same locations in the 3D space. The sliding step is important as it places the landmarks in positions where they correspond better to each other across individuals [26]. The semi-landmarks were allowed to slide on the curves and surface mesh of each target using TPS warping of the template. This positions the reference points on the target facial mesh by minimizing the bending energy.

According to FL Bookstein [18], physical steel takes a bending form with a small displacement. This is because the function (*x*, *y*, *z*) is the configuration of lowest physical bending energy which is consistent with the given constraints. In this 3D face deformation, the transformation of TPS was done mathematically by interpolation of smooth mapping of *h* from *ℝ*^3^ → *ℝ*^3^ which is a selection of a set of corresponding points {Ρ_*Ri*,_Ρ_*Ti*_}, *i* = 1, …, *N* on the reference object (template) and target (subject) faces minimizing the bending energy function Ε(*h*) using the following interpolation conditions [7, 18, 63]:

$$ \mathrm{E}(h)={\iiint}_{{\mathbb{R}}^3}\Big({\left(\frac{\partial^2h}{\partial {x}^2}\right)}^{\mathbf{2}}+{\left(\frac{\partial^2h}{\partial {y}^2}\right)}^{\mathbf{2}}+{\left(\frac{\partial^2h}{\partial {z}^2}\right)}^{\mathbf{2}}+ $$

4 $$ 2{\left(\frac{\partial^2h}{\partial xy}\right)}^{\mathbf{2}}+2{\left(\frac{\partial^2h}{\partial xz}\right)}^{\mathbf{2}}+2{\left(\frac{\partial^2h}{\partial yz}\right)}^{\mathbf{2}}\Big) dxdydz $$

$$ s.t.\kern1.75em h\left({\mathrm{P}}_{Ti}\right)={\mathrm{P}}_{Ri},i=1,\dots, M $$

where Ρ_*Ti*_ is the target object and Ρ_*Ri*_ is the reference object of the sets of corresponding points, *h* is the bending energy function that minimizes non-negative quantity of the interpolation of the integral bending norm or the integral quadratic variation Ε(*h*). TPS now form a decomposition of each component into affine and non-affine components such that,

5 $$ h\left({\mathrm{P}}_h\right)=\Psi \left({\mathrm{P}}_h\right)\mathrm{K}+{\mathrm{P}}_h\Gamma $$

where Ρ_*h*_ is the homogeneous coordinate points on the target 3D face, and Ψ(Ρ_*h*_) = (Ψ_1_(Ρ_*h*_), Ψ_2_(Ρ_*h*_), …, Ψ_*M*_(Ρ_*h*_)) is a 1 × M kernel vector of TPS with the form:

6 $$ {\Psi}_w\left({\mathrm{P}}_h\right)=\parallel {\mathrm{P}}_h-{\mathrm{P}}_{Tw}\parallel $$

while Κ is a M × 4 non-affine warping coefficient matrix, and Γ is homogeneous affine transformation of 4 × 4 matrix. The energy function is minimized to find optimum solution in (4) if the interpolation condition in (1) is no longer necessary.

7 $$ \mathbbm{E}\left(\beta, K,\Psi \right)=\frac{1}{M}{\sum}_{J=1}^M\parallel h\left({\mathrm{P}}_{Tj}\right)-{\mathrm{P}}_{Rj}\parallel +\beta E(h) $$

The interpolation conditions in (1) are satisfied if the smoothing regularization term *β* is zero; Γ and Κ are TPS parameters obtained by solving the linear equation:

8 $$ \left(\begin{array}{cc}\Psi & {\mathrm{P}}_R\\ {}{\mathrm{P}}_R^T& 0\end{array}\right)\left(\begin{array}{c}K\\ {}\Gamma \end{array}\right)=\left(\begin{array}{c}{\mathrm{P}}_T\\ {}0\end{array}\right) $$

Ψ is a M × M matrix with the component Ψ_*wl*_ =  ∥ Ρ_*Tw*_ − Ρ_*Tl*_ ∥ *and* Ρ_*R*_ is a M × 4 matrix with each row being the homogeneous coordinate of the point Ρ_*Ri*_, *i* = 1, …, *M*. Using (2), the target facial mesh Ρ_*Ti*_ is deformed to the reference mesh Ρ_*Ri*_. Applying the bending energy, the process was iterated specified number of cycles (6) to have optimum sliding of the points on the facial surface which gives points relaxed. This changed the bending energy from initial value *E*_*i*_ to final value *E*_*f*_ after a complete iteration. This makes the semi-landmarks to be treated the same as homologous landmarks with respect to downstream analyses. Because the warping may result in points that do not lie directly on the facial surface on the target mesh, the transferred points were projected on the closest point on the mesh surface. This was done using Iterative Closest Point (ICP) method [8], which aims to iteratively minimize the mean square error between two point sets. If the distance between the two points is within the acceptable threshold, then the closest point is determined as the corresponding point [64]. The homologous landmark warping *H*_*K*Γ_ after a six complete iterations is, therefore:

9 $$ {H}_{K\Gamma}={E}_{f-i}\left(\begin{array}{c}K\\ {}\Gamma \end{array}\right) $$

**Fig. 6:**
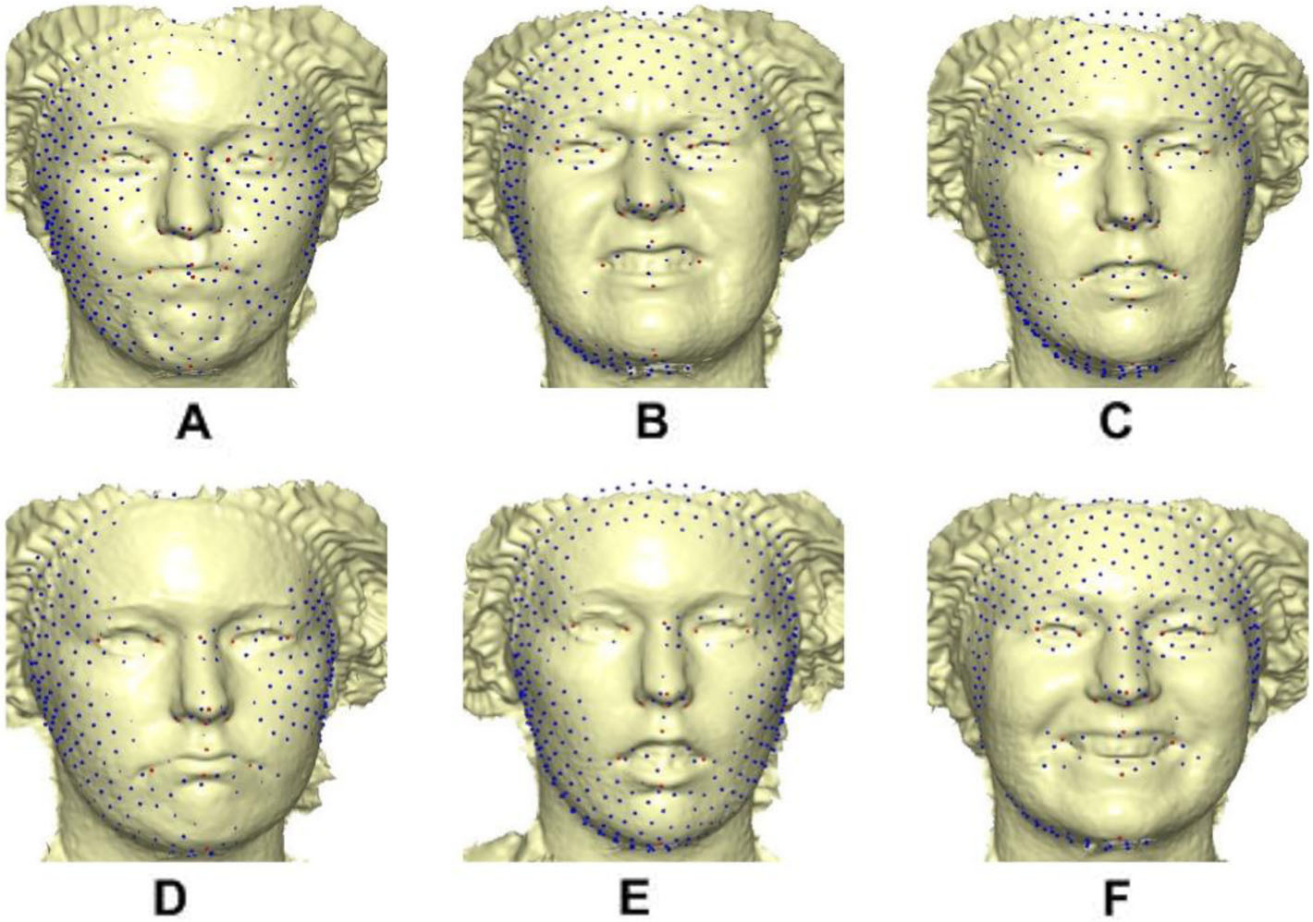
Partially warped 500 sliding point on target facial surface. **a** Angry. **b** Disgust. **c** Fear. **d** Sad. **e** Surprise. **f** Happy

**Fig. 7:**
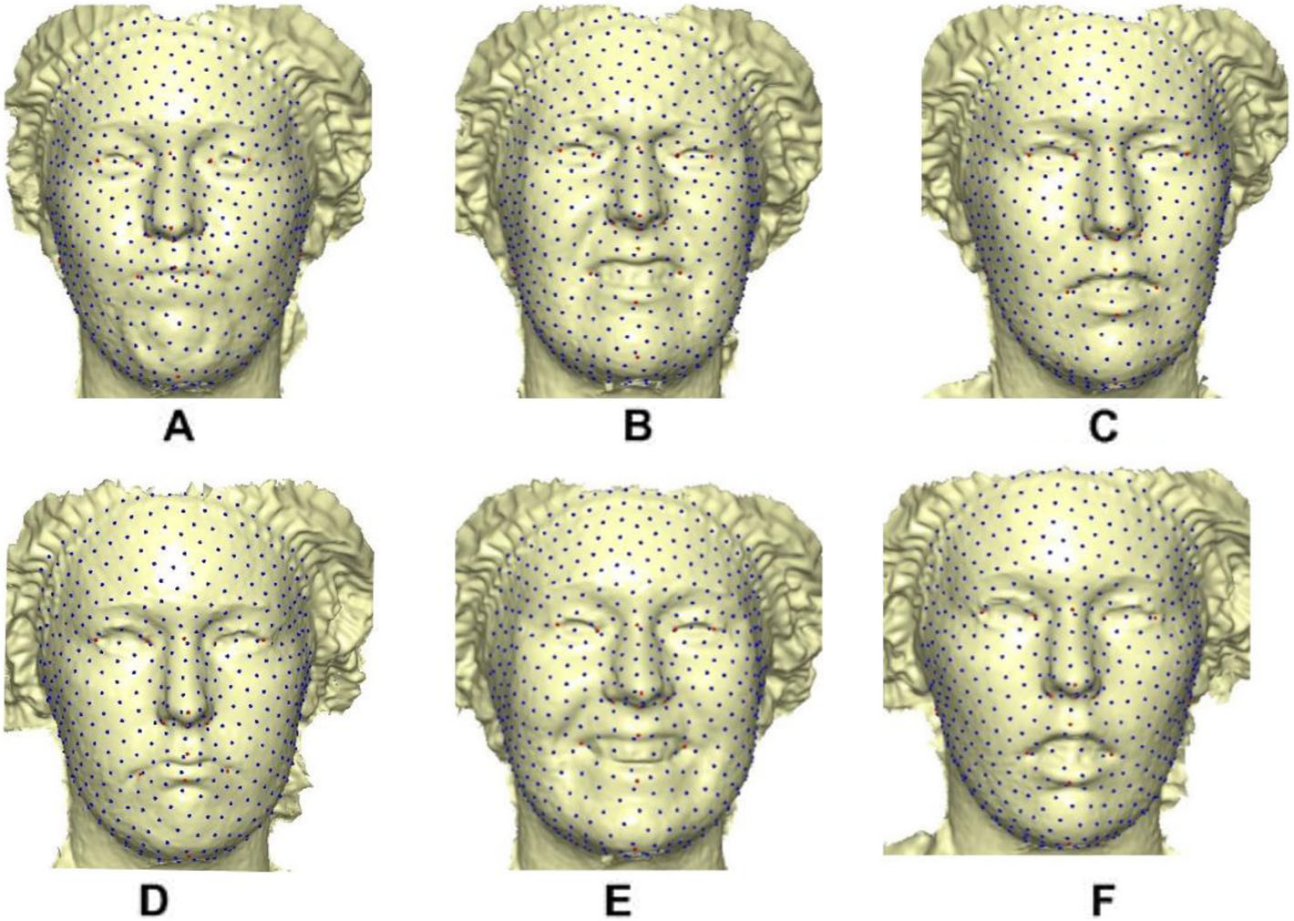
Complete and homologous 500 warped points on target mesh. **a** Angry. **b** Disgust. **c** Fear. **d** Sad. **e** Surprise. **f** Happy

Where

10 $$ \left(\begin{array}{c}K\\ {}\Gamma \end{array}\right)={\left(\begin{array}{cc}\Psi & {\mathrm{P}}_R\\ {}{\mathrm{P}}_R^T& 0\end{array}\right)}^{-1}\left(\begin{array}{c}{\mathrm{P}}_T\\ {}0\end{array}\right), $$

is the linear TPS equation obtained during deformation surface of the target mesh to the reference mesh before convergence was finally reached and *E*_*f* − *i*_ = *E*_*f*_ − *E*_*i*_ of six complete iterations. The first iteration showed a partial distribution of sliding points on the target surface mesh (Fig. 6). This was automatically repeated until optimum homologous result was achieved using exponential decay sliding step of hundred to 5 %. During the relaxation of the spline, the semi-landmarks slid along the surface and the curve tangent structures, and not on the surfaces or the curves which reduced the computational effort. This makes the minimization problem become linear, as sliding along the tangents lets the semi-landmarks slip off the data [22]. The target surface mesh is now treated as homologous points (Fig. 7). Note that we did not build a new deformable mathematical equation from scratch but extended the standard deformable method that has been established in [7].

In assessing error, 18 subjects (three from each expression) from each dataset were randomly selected; each one belonging to a different individual, distinct from the template subject. Each was digitized twice following the same method to account for digitization error. The results were analyzed using Procrustes ANOVA [65, 66] which has been implemented in morphometrics to analyze measurement error in MorphoJ [67–69]. This is done by the minimization of the squared sum of the distance of all objects and the consensus configuration [51].

### Feature selection with PCA

The features were selected by dimensionality reduction using Principal Components Analysis (PCA). Here, the data is represented as matrix *M* = [*m*_1_, *m*_2_, …*m*_*n*_], where *m*_*i*_ is the *ith* column vector representing the *ith* training data. The covariance matrix *K* =  *cov* (*M*) = *MM*^*T*^, we then carried out eigenvalue decomposition on the matrix *M* to produce highest ranking eigenvectors known as Principal Components (PCs) with the help of their corresponding eigenvalues. We chose *x* eigenvectors (*p*_1_, *p*_2_, …, *p*_*n*_) that best described the data with projection onto the space spanned by these vectors such that $X={\left[{p}_1,{p}_2,\dots, {p}_n\right]}^{T_m}$; where *X* is the *n* dimensional vector used as features during the training and classification process. The total PCs computed during reduction process is 239PCs and 179PCs for Stirling and Bosphorus, respectively (see Additional file 3: Table S3 and Additional file 4: Table S4). Among these, only 135PCs from Stirling and 98PCs from Bosphorus which have been observed to have the highest ranking eigenvectors were selected for classification using Bartlett’s test for the first principal component method [70, 71]. In other to establish total PCs that expressed meaningful variation in each expression group, a broken stick was used [70, 72]. This is based on the eigenvalues from random data of the principal components.

### Linear discriminant analysis (LDA)

The method used multi-class LDA to classify the features. This is one of the supervised learning methods for classification. It operates by maximizing the ratio of between-class variance to that of within-class variance in a dataset, thereby guaranteeing maximum separability. It has been widely applied to many applications such as microarray data classification [73], face recognition [74], and image retrieval [75]. LDA comes with singularity problem [76] which has given room to many extensions to LDA such as regularized LDA [77], pseudo-inverse LDA [78], and subspace LDA [79]. In order to overcome the singularity issue of classical LDA, PCA was applied as an intermediate dimensionality reduction.

Computing LDA for multi-class is slightly different from two-class. The multi-class requires the application of multiple discriminant analysis [80]. The maximization of ratio of within-class scatter to between-class scatter is done among the competing classes [81]. The multi-class can also be called Canonical Variates Analysis (CVA) but the major assumption for LDA is that the variance–covariance matrices are all equal [82]. To simplify the computational process, we first computed the within-class matrix for n classes (*n* = 6 for this study) such that:

11 $$ {\hat{\Sigma}}_w={S}_1+\dots +{S}_n={\sum}_{i=1}^n{\sum}_{\mathrm{X}\in ci}\left(X-{\overline{X}}_i\right){\left(X-{\overline{X}}_i\right)}^{\prime } $$

followed by between-class matrix, given by:

12 $$ {\hat{\Sigma}}_b={\sum}_{i=1}^n{m}_i\left({\overline{X}}_i-\overline{X}\right){\left({\overline{X}}_i-\overline{X}\right)}^{\prime } $$

where *m*_*i*_ is the number of samples for each class, ${\overline{X}}_i$ is the mean vector for each class and *X* is the summed mean vector computed as $\overline{X}=\frac{1}{m}{\sum}_{i=1}^n{m}_i{\overline{X}}_i.$

By obtaining the within-class and between-class matrices (${\hat{\Sigma}}_w$ and ${\hat{\Sigma}}_b\Big)$, we now obtained the transformation Φ by solving generalized eigenvalue problem:

13 $$ {\hat{\Sigma}}_b\Phi =\lambda\ {\hat{\Sigma}}_w\Phi $$

Once the transformation Φ is solved, the classification is then performed based on distance metrics in transformed space. Here, Euclidean distance is applied such that:

14 $$ d\left(x,y\right)=\sqrt{\sum_i{\left({x}_i-{y}_i\right)}^2} $$

and cosine measure

15 $$ d\left(x,y\right)=1-\frac{\sum_i{x}_i{y}_i}{\sqrt{\sum_i{x}_i^2\ {y}_i^2}}\kern0.5em , $$

we arrive at a new instance $\mathbbm{z}$, which classified into $argmin\ d\left(\mathbbm{z}\Phi, {\overline{X}}_k\ \Phi \right),\kern0.5em$ where ${\overline{X}}_k$ is the centroid of k-th class. The advantage of multiple discriminant analysis over single discriminant analysis is that it produces an elegant classification with the use of discriminant features [81].

## Supplementary information

**Additional file 1: Table S1.** Raw three-dimensional digitized data for Stirling dataset expression group.

**Additional file 2: Table S2.** Raw three-dimensional digitized data for Bosphorus dataset expression group.

**Additional file 3: Table S3.** PCs scores for all subjects used in the Stirling dataset.

**Additional file 4:.** **Table S4.** PCs scores for all subjects used in the Bosphorus dataset

## Data Availability

Raw three-dimensional digitized data for each expression group and principal component analysis scores of the entire subjects. Table S1 and S2: Three-dimensional raw data for each expression group in different sheet for Stirling and Bosphorus, respectively. Table S3 and S4: PCs scores for all subjects used in the experiment for Stirling and Bosphorus, respectively. Note that the 3D face dataset is not permitted to be shared by third party according to the license agreement.
